# Recognition of brief sounds in rapid serial auditory presentation

**DOI:** 10.1371/journal.pone.0284396

**Published:** 2023-04-13

**Authors:** Merve Akça, Jonna Katariina Vuoskoski, Bruno Laeng, Laura Bishop

**Affiliations:** 1 RITMO Center for Interdisciplinary Studies in Rhythm, Time and Motion, University of Oslo, Oslo, Norway; 2 Department of Musicology, University of Oslo, Oslo, Norway; 3 Department of Psychology, University of Oslo, Oslo, Norway; IRBA (French Armed Forces Biomedical Research Institute), FRANCE

## Abstract

Two experiments were conducted to test the role of participant factors (i.e., musical sophistication, working memory capacity) and stimulus factors (i.e., sound duration, timbre) on auditory recognition using a rapid serial auditory presentation paradigm. Participants listened to a rapid stream of very brief sounds ranging from 30 to 150 milliseconds and were tested on their ability to distinguish the presence from the absence of a target sound selected from various sound sources placed amongst the distracters. Experiment 1a established that brief exposure to stimuli (60 to 150 milliseconds) does not necessarily correspond to impaired recognition. In Experiment 1b we found evidence that 30 milliseconds of exposure to the stimuli significantly impairs recognition of single auditory targets, but the recognition for voice and sine tone targets impaired the least, suggesting that the lower limit required for successful recognition could be lower than 30 milliseconds for voice and sine tone targets. Critically, the effect of sound duration on recognition completely disappeared when differences in musical sophistication were controlled for. Participants’ working memory capacities did not seem to predict their recognition performances. Our behavioral results extend the studies oriented to understand the processing of brief timbres under temporal constraint by suggesting that the musical sophistication may play a larger role than previously thought. These results can also provide a working hypothesis for future research, namely, that underlying neural mechanisms for the processing of various sound sources may have different temporal constraints.

## Introduction

Beyond merely being a perceptual attribute of sound, timbre is known to play a primary role in the recognition, categorization, and identification of sound-producing events or sound sources when these sources are outside of the field of vision [1–3]. Converging evidence suggests that from quick evaluations about the source of the sound (e.g., [4–6]), to identifying a familiar person from their voice (e.g., [7]) or categorizing or identifying a music genre (e.g., [8, 9]), healthy listeners are remarkably efficient at recognizing and identifying sounds. Anecdotally, people can also skillfully recognize and attend to sounds in the midst of rapidly changing auditory scenes in everyday life. Drawing from this human capacity, in this article, we studied the role of musical sophistication, working memory capacity, and sound duration on timbre recognition under the temporal constraints of rapid presentation streams.

### Recognizing timbre in short sound segments

Handel [1] postulated that cues for timbre are context-dependent, meaning that they vary across many contextual factors, including sound duration. Other factors include frequency, intensity, the comparison sound stimuli, and task structure. The temporal evolution of the sound provides useful cues for timbre perception, as the acoustic information enabling source identification develops over time. An intriguing question following this concerns what happens to this remarkable human ability when the duration is severely reduced and held constant across different timbres. Research on timbre recognition in brief sounds in humans is essential for our understanding of the temporal aspects of auditory processing. In experimental paradigms that require the recognition of brief targets, presented among a series of distractor stimuli, timbre is the most applicable sound parameter to vary. Timbre can represent auditory objects and learned categories such as musical instruments, voices, or environmental sounds (e.g., [1]), while the perception of pitch and loudness is, in comparison, more relative due to their unidimensionality (e.g., [10]).

Previous studies have revealed that listeners can recognize timbre with above chance level accuracy under remarkably brief sound duration thresholds. In his seminal paper, Gray [11] compared vowel identification performances of listeners under durations varying from 3 to 520 ms and found that the listeners reached an above chance level performance already at 3–4 ms short vowel segments. Since then, a growing body of research demonstrates human auditory perception capacity under fascinatingly short duration thresholds, though only a few of these explorations included non-speech sounds. In those studies where more extended sound sources were used, the duration thresholds were short but varying.

For brief musical instrument sounds (i.e., single notes from brass, flute, harpsichord or string instruments), Robinson and Patterson [12] demonstrated that independent of the pitch chroma cues, timbre of musical instrument sounds can be reliably identified with above chance level accuracy for durations as short as a single cycle of a waveform. Furthermore, early research on musical instrument identity showed that segments of a note can yield enough acoustic information for sound source identification (for a summary of those studies, see [1]).

Aiming to find the minimum duration supporting auditory categorization, more recently Bigand and colleagues [13] tested participants with no musical training with sound segments from 20 to 200 ms belonging to the three different categories (voice, musical instruments, environment sounds). They found that 50 ms sound segments were sufficient for categorization of spoken voices, instrumental sounds and environmental sounds above chance level. For voices and musical instruments, above chance level performance started to be seen already at 20 ms segments. With fewer variations in the sound set, Suied and colleagues [14] provided an intriguing demonstration suggesting that as little as a few milliseconds (4 ms for voices, 8 ms for instruments) allows for above chance level categorization of a single sound belonging to a predefined target category (e.g., sung voices, percussion, strings). This result is surprising, as one would expect that with more limited acoustic information, that is, when the judgement was based solely on timbre cues, one would need longer stimuli duration for successful categorization. These differences in minimum sound duration are also likely due to other contextual factors, such as the task (e.g., type of categorization), learning effects, and participant factors (e.g., differences in *musical sophistication*– that is, “a psychometric construct that can refer to musical skills, expertise, achievements, and related behaviours across a range of facets” [15]).

### Effects of sound source in recognizing timbre from short sounds

Notably, several lines of research seem to indicate that constraints on sound duration may affect the processing of certain sound sources differently than others. In particular, human voices were often reported as having an advantage, both in the way that they were categorized above chance at lower duration thresholds than instrumental music (e.g., [14, 16]) and environmental sounds (e.g., [13] but for conflicting evidence see also the results of the same study under the peak-normalization condition), and also in the way that they were recognized faster under speeded recognition tasks where inhibition of response was required for instrument distracters [6]. In this paper, we sought to investigate whether the voice advantage would be present in a presentation context that is different than the previous investigations. We contrasted voices with cello tones due to their shared perceptual similarities with human voices (e.g., [17, 18]), with pure tones, as they do not have the complexity of voice and musical instrument tones, and with bell tones, as they semantically belong to the environmental sound category but are more musical.

Other than speech and music, environmental sounds constitute a vast majority of the sounds around us. Given the prevalence of environmental sounds, often acting as ‘distracters’ in modern everyday life, we decided to present the chosen target sounds in the context of the environmental sounds, though this is not an attempt to make the sound stimuli ‘ecologically valid’ (as this is not reasonable to expect given the stimuli are very brief). This is a step towards understanding recognition amongst a wide range of sounds under duration constraints. Linking these lines of work, in this article, we examine recognition for brief sounds with different timbral qualities presented among environmental sounds in a rapid pace.

### Rapid serial stimulus presentation

Rapid serial stimulus presentation experiments, in which participants have the task of identifying or detecting one or more target at the end of the presentation stream, have been a useful way of exploring the temporal characteristics of perceptual and attentional processes [19]. Rapid serial presentation is not a newly discovered paradigm, but only a few studies have applied this paradigm, commonly used in the visual modality, to the auditory field. Analogous to rapid serial visual presentation (RSVP), the rapid serial auditory presentation (RSAP) paradigm also enables us to study the rate at which the human auditory system can reliably process and differentiate a series of sounds.

The method was first introduced by Mary C. Potter (e.g., [20]) for studies of visual cognition. It has been applied often in the study of attention (e.g., [21]) and to investigate the phenomena of the attentional blink or repetition blindness (e.g., [22]). The rapid serial stimulus presentation method has proven invaluable also in neuroscience studies. One application is in electrophysiological studies of visual neurons’ selectivity in the cortex of monkeys (e.g., [23]), where the method provides the advantage of presenting a large variety of visual stimuli in a short period of time. The method has been consequently applied to non-invasive recordings using either EEG or MEG to specifically study human vision (e.g., [24, 25]). Although the method was initially used as a visual paradigm, it has revealed to be relevant also for the investigation of either neural processing within the auditory modality as the rapid serial auditory presentation (RSAP) or even with multimodal, audiovisual, simultaneous processing (e.g., [26]). Most of the studies that have used RSAP have focused on speech processing (like the perception of syllables, e.g., [27]). However, a few EEG studies have used rapid serial presentation of non-verbal sounds like simple tones varying in either frequency or amplitude (e.g., [28]). Wider utilization of rapid serial presentation paradigms would greatly advance our understanding of the neural underpinnings of auditory attention and recognition. To our knowledge, no study within the neurosciences has applied the RSAP to sounds of different timbre. This is remarkable, given that the timbre is analyzed early within the auditory system, given that animal studies show that neurons in the inferior colliculus (i.e., the first processing station in the brain to receive input from the ear) encode flux in timbre (e.g., [29]). Neuroimaging studies in musicians confirm that the inferior colliculus processes spectrotemporal acoustic properties like roughness and flux [30]. Hence, RSAP combined with neuroscience methods may throw light on auditory processing from the early stages to higher in auditory processing. In addition, timbre information uniquely identifies categories or even exemplars of natural kinds (e.g., the type of object or organism producing a sound or of a specific individual, like the voice of a person). Hence like color or form in vision, timbre is a fundamental property of the brain’s perceptual analysis and recognition of world objects.

In the majority of the studies on timbre recognition in short sound segments, the brief complex sounds were presented in isolation. Presenting single isolated sounds and sounds among RSAP provide different contexts for studying timbre recognition at brief durations. The duration thresholds identified by the previous studies where a single sound presented in isolation, as interesting as they can be, do not necessarily represent the thresholds for recognition among more complex stimuli. This is because processing a briefly presented single sound in isolation is going to be markedly easier than processing the same target presented among a stream of sounds (for an analogous reasoning in the visual domain, see [31]). For a better picture of the processing rate and duration threshold estimations for auditory recognition, more studies using RSAP paradigm are needed. To the best of our knowledge, thus far only two studies have applied RSAP paradigm to estimate the processing rate of timbre recognition. First, Suied and colleagues [32] introduced the RSAP paradigm combined with a timbre recognition task, where the participants were to indicate whether each sequence contained a voice or not (the distracters in the sequence were musical instrument sounds). Their pilot data showed that listeners can recognize voice targets above chance level at rates up to 30 sounds/second (approx. 33 ms per item).

More recently, Isnard and colleagues [16] further investigated the processing speed needed for voice and instrument targets when presented among opposite category distracters under four experiments with various test conditions (i.e., pitch, sound duration, comparison of fixed number of sounds and fixed duration in a sequence). Their results indicated not only that the voices among instrument sounds were recognized faster and better than the instrument sounds among voices, but also that, for all experimental conditions, auditory recognition can be extremely fast. As in Suied et al. [32], their participants could recognize targets embedded in the sound sequence above chance level for up to a 30 Hz presentation rate, corresponding to 33 ms stimulus duration per item. Even in the shortest sound duration they tested (16 ms) recognition was above chance level. In both these RSAP studies, however, the participant selection criteria was set on a prior experiment where participants were tested on their ability to recognize brief sounds (varying from 2 to 128 ms) presented in isolation. Given that participants were not naïve to the sounds presented in the RSAP paradigm, the recognition performance found in these investigations are likely to be higher and/or duration thresholds briefer (for above chance level performance) than they would otherwise.

### Effects of musical sophistication on timbre recognition

Differences in the musical sophistication level of the participants can be of a particular relevance in the quest to identify the lowest duration threshold that allows for timbre recognition. However, with the exception of Bigoni and Dahl [33], the current literature lacks explorations of the relationship between musicality and brief sound recognition based on timbre. In their study, Bigoni and Dahl tested whether musicians’ thresholds for timbre discrimination would be lower than that of non-musicians [33]. Their results showed an overall high task performance, with no statistically significant difference between musicians and non-musicians. As the authors also discussed, this might be partly explained by the criteria used for participant assignment to musician and non-musician groups, which was based on the years of formal training and/or performance experience (with a cutoff point of 5+ years). An alternative approach would be to quantify the musical sophistication of the participants.

Musical sophistication gives a comprehensive indication of how actively and capably people engage with music. This multifaceted construct can be measured in the general population, including in individuals who consider themselves non-musicians [15]. It is beneficial to address the relationship between musical sophistication and timbre recognition from brief sounds using a measure that is able to capture the wide range of effects that can be readily present even within the non-musician group. This way, we are able to capture the potential effects of musical sophistication that do not necessarily arise strictly from theoretical knowledge of or professional involvement in music-making.

We measured musical sophistication with the general musical sophistication factor of Goldsmith Musical Sophistication Index (Gold-MSI; [15]). This index draws of different facets of musical engagement, including active engagement (e.g., listening habits), perceptual abilities, singing abilities, formal training, and emotional engagement with music. The self-report inventory of Gold-MSI has a high test-retest reliability (0.88 to 0.97; [15]) and a good internal consistency for the general musical sophistication factor (Cronbach’s alpha = 0.93; [15]). The scores in the General Musical Sophistication sub-scale range between 18 and 126, and higher scores are assumed to reflect higher levels of musical sophistication.

Defined as a brain system dedicated to temporarily maintaining and storing information necessary for complex cognitive activities [34, 35], working memory (WM) might also affect timbre recognition. The RSAP paradigm that was used in the present study invites participants to hold a target sound in memory temporarily, that is, until the auditory presentation stream is complete and the response can be given. Prior research has linked working memory load or capacity with listeners’ success at selectively attending to relevant auditory information [36, 37]. Listeners are less able to maintain non-verbalizable timbral information in working memory than verbal information like digits, resulting in a reduced WM capacity for timbre [38]. Retention is better for familiar timbres of acoustic instruments than for unfamiliar, digitally-transformed timbres [39]. Similarly to the word similarity effect that has been observed for verbal WM, acoustically-similar timbres are more difficult to retain in WM than acoustically-dissimilar timbres. In the present study, we used a verbal WM assessment to investigate the potential link between timbre recognition and WM capacity.

### Present study

The present study builds on and extends the previous works summarized above on timbre recognition in brief sounds. The aim of the present experiments was to test the effects of participant factors (i.e., musical sophistication and WM capacity) and stimulus factors (i.e., sound duration and timbre) on auditory recognition by matching the sound to a target sound category in a rapid auditory presentation stream of brief sounds.

Using an established paradigm of rapid serial presentation, here we tested the recognition for brief sounds in a novel context. That is, we examined whether individual differences in musical sophistication (Experiment 1a & 1b) and working memory capacity (Experiment 1b) could be related to performance on brief sound recognition task. In the present study, individuals’ musical sophistication levels were quantified using Gold-MSI [15]. We expected a positive correlation between the musical sophistication scores and performance in auditory recognition task. We also wanted to explore whether higher musical sophistication scores could be associated with recognition of sounds in challenging listening environments, i.e., low duration thresholds and rapid presentation rates.

In addition to the exploratory examinations of the factors mentioned above, we ask the following research question: When searching among environment sounds, are certain sound sources (e.g., voices) recognized at shorter durations than other sounds (e.g., instrument tones, pure tones, etc.)? If there is a recognition advantage for one or more type of sound source(s) under shorter duration constraints, this could suggest that the underlying mechanisms for the processing of a given sound source may have different temporal constraints.

Parallel to the differences amongst the various timbres in terms of familiarity and sound quality, we expected participants’ ability to recognize targets selected from various sound sources to differ. In particular, consistent with the voice advantage reported in the literature, we hypothesized that the recognition for voice targets will be better than the rest of the target categories. We also hypothesized that the recognition for the bell targets to be the worst, as the bells are both semantically and acoustically more similar to the distracters (i.e., environmental sounds) in this study. Furthermore, we expected a linear decrease in recognition sensitivity as the duration decreases.

Additionally, we investigated whether participants’ level of subjective certainty (or confidence), reaction times (Experiment 1b) correlate with their overall performance on the single target recognition task embedded in a RSAP paradigm with brief stimuli.

## Materials and methods

### Experiment 1a

#### Participants

Twenty-four volunteers (mean age: 25.92 ± 3.83, 15 females) participated in this study. All participants reported normal hearing and had either very little or no formal musical training. The average general musical sophistication score of the participants was 58.37 ± 13.69, which ranked at the 14th percentile based on 147,633 participants from Müllensiefen et al. [15]. The participants provided written informed consent and received a gift card worth 100 NOK for their participation. The experiments conformed to the Helsinki Declaration and to the national ethical guidelines for experiments with human subjects. A power analysis was run to determine the required sample size using G*Power [40]. Given a medium effect size ($\eta_{p}^{2}$ = .06), a minimum of 11 participants were required to achieve a power of 80% but we tested 24 participants to reach a full counterbalancing.

#### Stimuli

The stimuli were brief sounds belonging to different categories (160 environment sounds, 12 human voice, 12 cello tones, 12 sine tones, and 12 bell sounds). Cello and bell sounds (i.e., tubular bell) were sampled from the McGill University Master Samples DVD Set [41], voices were sampled from The Berklee College of Music Sampling Archive Vol. 5 [42], and the rest of the stimuli were sampled from freesound.org. Environmental sounds included sounds of everyday objects (e.g., printer, motorbike). Human voices comprised recordings of vowels sung by a female speaker. Female voice was selected to include mid-range pitches. In the experiment, environment sounds served as distracters, while the voices, sine tones, and bell sounds were targets. Detailed descriptions of timbre-related acoustic features of each stimulus category are shown in S1 Appendix in S1 File.

The targets were selected at 3 different pitches (A4–440 Hz, F4–349Hz, D♯4–331Hz). For voice targets, this means that one voice was presented at three pitches. All stimuli were normalized using the peak normalization method (i.e., an audio adjustment technique based on highest level of signal present in the waveform) and truncated to 60, 90, 120, and 150 milliseconds from the quasi-stationary portions of the sounds. Peak normalization, as opposed to root mean square (RMS) method, was opted for since RMS normalization could bias better voice detection (see findings of Bigand and colleagues [13] for a comparison of the RMS and peak normalization methods). They all included 2 milliseconds linear amplitude ramps to eliminate the onset and the offset clicks.

#### Procedure and design

The experiment was conducted at the cognitive laboratory of the Psychology Department at the University of Oslo. E-Prime 3.0 (Psychology Software Tools, Pittsburgh, PA, USA) running on a Windows PC was used to program the experiment, to deliver the auditory stimuli through headphones (Beyerdynamic DT770 Pro) and to collect participant responses. Participants were seated comfortably in a distraction-free, quiet room in front of a screen and completed a single-target recognition task within the RSAP paradigm. In the task, participants were asked to listen to a rapid stream of brief sounds and later report whether a previously heard target sound was present or absent in the stream. Hence, recognition here means that listeners could match a sound to a target, which is different from identification (e.g., naming a sound as a cello).

Each trial began with the auditory presentation of a target sound in isolation, followed by a fixation cross (+) displayed in the center of the screen for 250 milliseconds. The target sound was either a voice, a cello tone, a pure sine tone or a bell sound. The participants were then presented with the rapid serial auditory stream of 20 sounds (with 10 milliseconds inter-stimulus interval in between) binaurally. Each stream consisted of one target (or none) and 19 (or 20) distracter sounds. There was neither a practice phase nor response feedback.

At 66.6% of the trials a target sound was present and at 33.3% of the trials the target sound was absent. When present, the target appeared either at the 5th or 15th temporal position in the stream to compare target recognition at an early and a late temporal position. Subjects were to respond, as quickly as possible but without compromising accuracy, whether the target sound was present or absent in the sound stream by pressing the “1” or “2” on keyboard respectively. At the end of a trial, they were asked to rate how certain they are about their choice on a 6-point Likert type scale ranging from “Very uncertain” to “Very certain”.

The experiment comprised of 144 trials divided into four blocks varying in duration (60, 90, 120, and 150 milliseconds). All participants went through all four blocks with the order being fully counterbalanced across subjects with 24 counterbalancing rounds. The presentation order of target type was randomized. In each experimental block, the distracters were randomly selected from the pool of environmental sounds in each duration condition. The order of the distracters varied randomly on every trial. A single testing session lasted around 40 minutes. The participants were also asked to answer a few demographics questions and fill part of the Gold-MSI musical sophistication questionnaire (the items relating to the general factor of musical sophistication) which is designed to measure musical sophistication in the general population [15].

#### Data analyses

For each subject and each condition, the trial outcomes were classified as hits, misses, false alarms or correct rejections according to the stimulus-response matrix of Signal Detection Theory.

A log-linear correction [43] was applied to all the hit and false alarm rates to correct for the extreme values of 1s and 0s. These were then used to compute the recognition sensitivity index (*d*’) using the following formula by Macmillian and Creelman [44]:

$$\begin{array}{c} d{\;}^{\prime }=z(Hit\;rate)-z(False\;alarm\;rate) \end{array}$$ (1)

A series of Repeated Measures analyses of variance (ANOVAs) were conducted on the *d*’ scores as well as the reaction times for Hits and on the subjective certainty reports of the participants using JASP [45]. The alpha level was set at 0.05 unless otherwise stated. Where the assumption of sphericity was violated, a Greenhouse-Geisser correction was applied. For the significant effects, post-hoc pairwise comparisons were conducted using Holm’s Bonferroni corrections.

Further Bayesian statistical analyses were computed to quantify evidence for the null and the alternative hypotheses. We used the default Cauchy prior of JASP (i.e., *r*-scaled fixed effects = 0.5 and for *t*-tests = 0.707) for the Bayesian analyses. 4 × 4 Repeated Measures Bayesian ANOVA yielded Bayes Inclusion Factor (BF*Inc*) across matched-models [46], which represents the evidence for all models that includes a certain effect compared to the evidence for equivalent models stripped of that effect. BF*Inc* was used for the reporting and interpreting the results, based on suggestions by Lee and Wagenmakers [47], adjusted from [48]. For more information on the Bayesian evidence ratio interpretations, see S2 Appendix in S1 File.

For the reported correlations, Pearson’s *r* correlation coefficient was used whenever possible and based on the nature of the hypotheses (i.e., to test a linear association, rather than a monotonic one). Correlations were reported with the Kendall’s tau coefficient when the test for bivariate normality was not assumed. All correlations are calculated from the average scores of each participant in order not to inflate the degrees of freedom.

### Results

#### Recognition sensitivity (*d*’)

**Fig 1:**
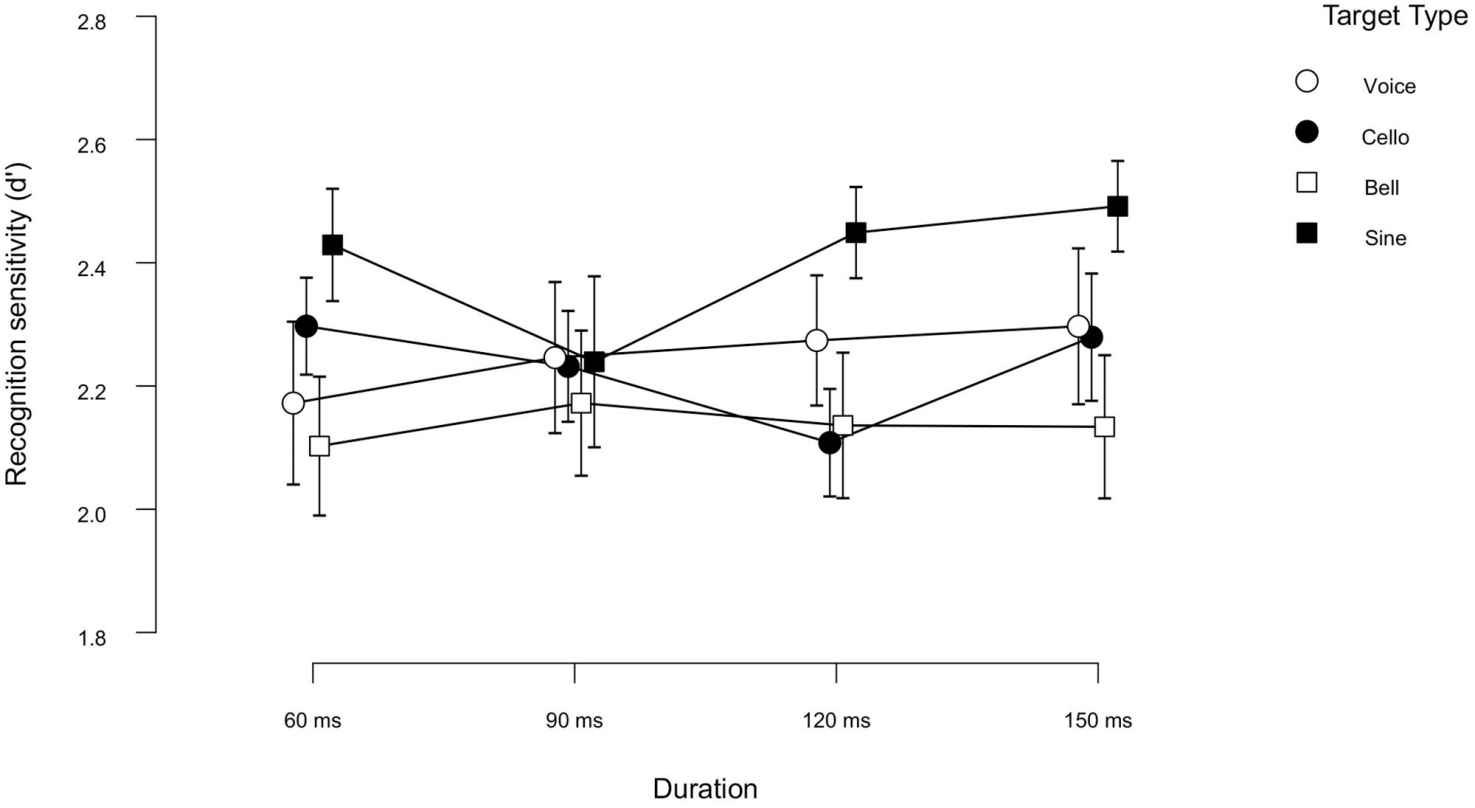
Target recognition sensitivity (*d*’) plotted as a function of duration (in Exp1a). Open circles, closed circles, open squares, and closed squares represent the data under the voice, cello, bell, and sine tone conditions, respectively. Error bars represent the standard error of the mean.

Fig 1 shows the sensitivity for recognition (*d*’) as a function of duration and target type. Overall, recognition sensitivity was well above the chance level (i.e., *d*’= 0) across all experimental conditions.

A 4 (Duration: 60, 90, 120, 150) × 4 (Target type: voice, cello, bell, sine tone) Within-Subjects ANOVA was conducted on the *d*’ scores. This analysis showed a significant main effect of Target Type [*F*(3, 69) = 4.47, *p* = 0.006, $\eta_{p}^{2}$ = 0.163]. Post-hoc contrasts revealed that participants were better at recognizing sine tones than the bell tones (Mean difference = -0.266, *SE* = 0.070, *p* = 0.004). No other significant difference was observed in the rest of the target type comparisons.

A Bayesian ANOVA showed moderate evidence for the main effect of Target Type over the null model (BF*Inc* = 3.321). The main effect of Duration was found non-significant [*F*(3,69)= 0.35, *p* = 0.79, $\eta_{p}^{2}$ = 0.015]. A further Bayesian analysis of variance was conducted to determine the evidence proportion supporting the null hypothesis, which indicated very strong evidence supporting the null model over the model with Duration (BF*Inc* = 0.018). The interaction of Duration × Target Type did not reach statistical significance either [*F*(5.203, 119.68) = 0.56, *p* = 0.74, $\eta_{p}^{2}$ = 0.024, Greenhouse-Geisser corrected]. The Bayesian ANOVA revealed very strong evidence in favor of the null model over the model with Target Type × Duration interaction (BF*Inc* = 0.011).

Together, these results suggest moderate evidence for the effect of Target Type alone (due to the difference between sine tones and bell tones), and a very strong evidence against the effect of Duration and the combined effect of Target Type and Duration on target recognition sensitivity. This means that the participants seem to be more sensitive in recognizing the sine tone targets than the bell tones. Furthermore, accurate recognition of a single auditory target during rapid serial auditory presentation is highly likely to be independent of the Duration and the combined effect of Target Type and Duration, at least for the duration range and the type of stimuli we have tested here.

#### Reaction times

Only the reaction times (RTs) for correct responses were taken into account. Responses faster than 200 milliseconds (i.e., anticipation errors) were excluded from further analysis. This removed less than 0.8% of the total trials. For the slow RT outliers, we used 2*SD* cutoff point, as the variability in means were high [49].

**Fig 2:**
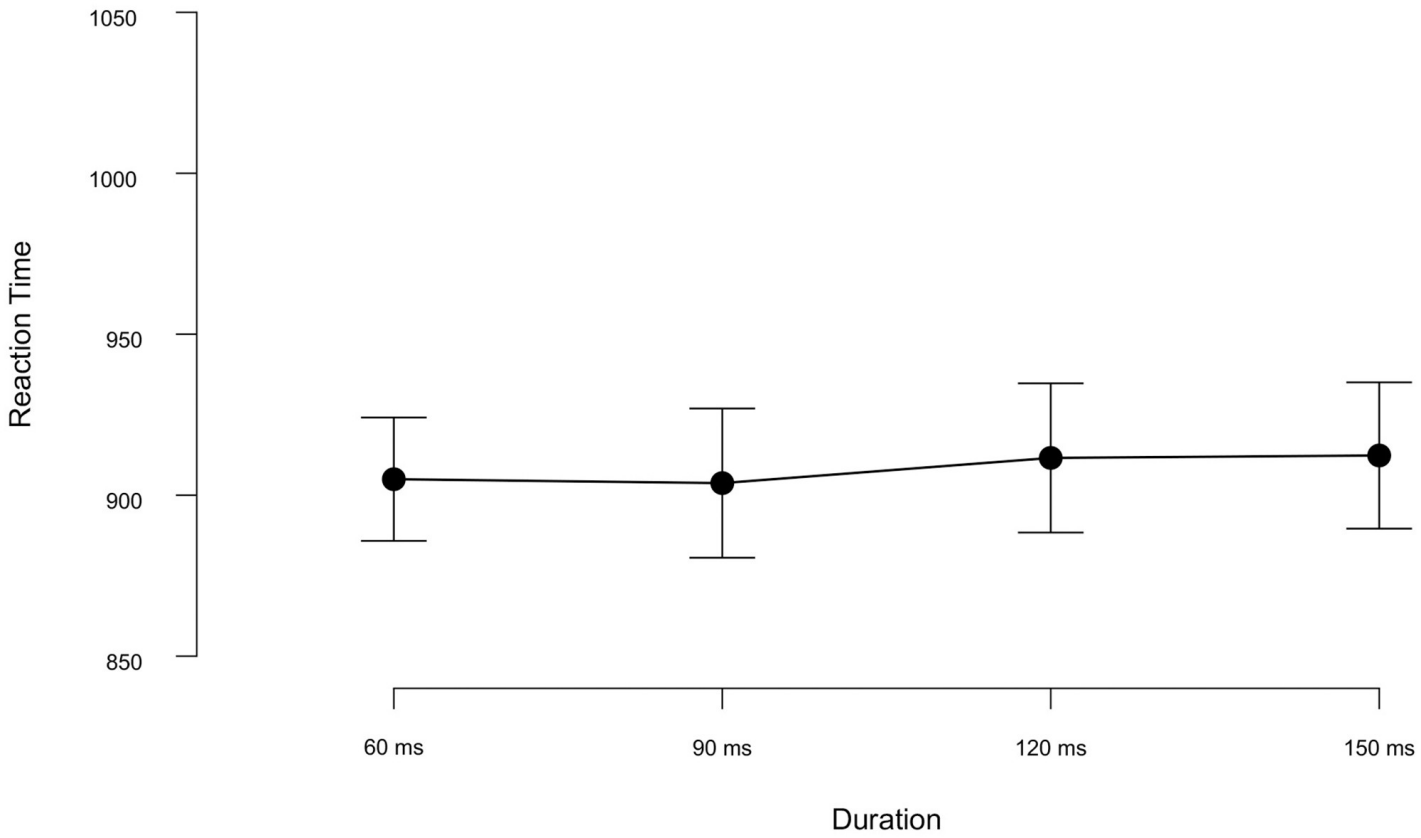
Reaction times (in ms) for target recognition as a function of stimulus duration for each item within each RSAP stream. Similar reaction times across all the duration conditions tested in Exp1a. Error bars represent the standard error of the mean.

As shown in Fig 2, the RTs for recognition were highly similar across RSAP streams with various stimulus durations (60—150 ms).

Unlike the results we obtained with the *d*’ sensitivity index, a 4 × 4 Repeated Measures ANOVA on RTs indicated a non-significant main effect of Target Type [*F*(3, 69) = 2.20, *p* = 0.10, $\eta_{p}^{2}$ = 0.087]. Neither the main effect of Duration [*F*(3, 69) = 0.01, *p* = 0.10] nor the Duration × Target Type interaction were significant [*F*(9, 207) = 1.02, *p* = 0.42, $\eta_{p}^{2}$ = 0.043]. Thus, none of our manipulations had a significant impact on the response times under 5% significance testing. We conducted a further Bayesian ANOVA to test the evidence proportions for these null results. It showed strong evidence against the main effect of Target Type (BF*Inc* = 0.045), very strong evidence against the main effect of Duration (BF*Inc* = 0.012) and extreme evidence against the Target Type × Duration interaction (BF*Inc* = 0.008). Hence, the analysis on the RTs conclusively supported the null model over the models with the Target Type and Duration alone as well as the combined effect of the two.

#### Level of subjective certainty

The participants’ subjective certainty ratings following their response for target recognition were overall high (*M* = 5.32, *SD*= 0.47).

A 4 × 4 Repeated Measures ANOVA on the subjective certainty levels showed a significant main effect of Target Type [*F*(2.199, 50.583) = 34.84, *p* < 0.001, $\eta_{p}^{2}$ = 0.602, Greenhouse-Geisser corrected], Duration [*F*(1.705, 39.211) = 9.23, *p* < 0.001, $\eta_{p}^{2}$ = 0.286, Greenhouse-Geisser corrected] and the Duration × Target Type interaction effect [*F*(3.992, 91.816) = 4.49, *p* = 0.002, $\eta_{p}^{2}$ = 0.163, Greenhouse-Geisser corrected].

Post-hoc comparisons revealed that the participants’ certainty ratings were significantly higher when the target was a sine tone than the other targets (Mean difference = -0.324 and *SE* = 0.042, *p* < 0.001 for voice vs sine, Mean difference = -0.324 and *SE* = 0.042, *p* < 0.001 for cello vs sine, Mean difference = -0.385 and *SE* = 0.042, *p* < 0.001 for bell vs sine tone). The certainty ratings were also significantly higher for targets in 90 ms than in 60 ms duration (Mean difference = 0.191, *SE* = 0.038, *p* < 0.001) and 150 ms (Mean difference = 0.140, *SE* = 0.038, *p* = 0.002). Finally, the difference in the certainty levels between sine tone targets and all other targets was the largest in the 120 ms condition. More specifically, in the 120 ms condition, the participants reported higher levels of confidence when the target was sine tone than voice (Mean difference = 0.616, *SE*= 0.073, *t*=8.402, *p* < 0.001), bell (Mean difference = 0.569, *SE* = 0.073, *t* = 7.770, *p* < 0.001, or cello (Mean difference = 0.407, *SE* = 0.073, *t* = 5.559, *p* < 0.001).

Additional analyses using the Bayes Factor indicated extreme evidence in favor of the models with Target Type (BF*Inc*= 1.202e+20), Duration (BF*Inc* = 1404.17) and the interaction of Target Type and Duration (BF*Inc* = 248.94). The model with Target type received the most support in explaining the data, which was 1.2 × 10^20^ times more probable than the null model.

#### Target position

No statistically significant difference was observed between targets appearing as the fifth versus the fifteenth item in the rapid serial auditory stream (*t*= 1.476, *p* = 0.153). A further Bayesian paired-samples *t*-test indicated anecdotal evidence against a difference between the two target positions (BF*01* = 1.793), meaning that the evidence was inconclusive to support this claim further.

#### Correlations

The musical sophistication scores of the participants did not correlate with the *d*’ scores, Hit rates, or False alarm rates (Pearson’s *r* = 0.12, *p* = 0.57; Pearson’s *r* = 0.32, *p* = 0.13; Pearson’s *r* = 0.02, *p* = 0.91, respectively).

**Fig 3:**
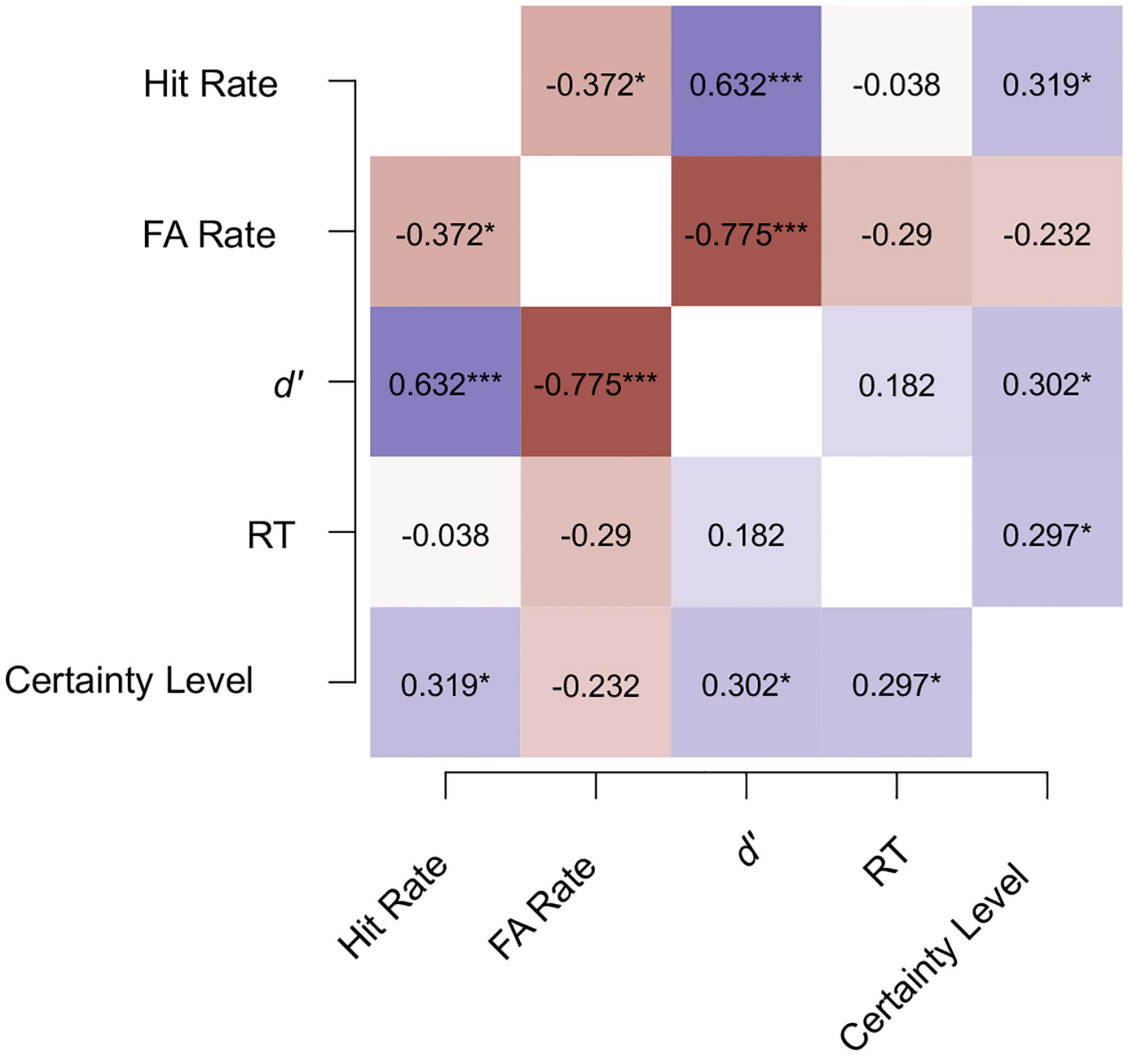
Heatmap of the correlations between the measures in Experiment 1a. The heatmap depicts Kendall’s tau correlations between the measures of Hit rate, False alarm (FA) rate, the sensitivity index (*d*’), reaction time (RT), and the subjective certainty level averaged for each participant. Blue and red colors correspond to positive and negative correlation coefficients, respectively. The saturation of colors illustrate the magnitude of the correlation coefficient. * *p* < 0.05, ** *p* < 0.01, ***, *p* < 0.001.

Correlations between the measures of Hit rate, False alarm rate, *d*’ sensitivity index, RTs, and the certainty levels are illustrated with a heatmap (see Fig 3). No significant correlations were observed between the *d*’ and RT data (Kendall’s tau = 0.18, *p* = 0.21) and between the RTs and the Hit rates (Kendall’s tau = -0.04, *p* = 0.80). There was a moderate positive association between the participants’ subjective certainty ratings following the recognition responses and the RTs (Kendall’s tau = 0.30, *p* < 0.05). Similarly, the relationship between the *d*’ scores and the certainty ratings was, too, positive and its magnitude was moderate (Kendall’s tau = 0., *p* < 0.05).

### Interim discussion

Experiment 1a established that the participants were successfully able to recognize brief target sounds well above chance level, with a d-prime of 2 to 2.6, under all experimental conditions. We found moderate evidence for the effect of Target Type on recognition sensitivity. Indeed, the participants were more reliable at recognizing sine tone targets than bell targets. However, this difference was not reflected in the RTs.

Despite very strong evidence reflecting that stimulus duration and the interaction effects were unlikely to impact recognition sensitivity, the participants’ subjective certainty ratings and comments pointed us in the direction that differences in WM capacity might have confounded these results. Although typically WM capacity is discussed in relation to the number of items to maintain in memory rather than in relation to duration, time-based limits on WM (e.g., memory traces to fade away over time) has been debated and challenged in the past following Baddeley and colleagues’ original study [50] on word-length effect, where longer duration words were recalled worse than shorter duration words. It remains unclear how the temporal duration interpretation of this speech-related effect may be relevant to processing streams of other (i.e., non-speech) sounds. In the present study, given that the total duration of the sound stream under different stimulus duration conditions varied as a consequence of the duration manipulation, it might have been more difficult to be maintain the representations for longer sounds in memory (and vice versa). We wanted to additionally test if there is a relationship between the WM capacity and recognition in the context of the present study. Should such a relationship exists, it would be useful to control for the individual differences in WM capacities. Thus, to be able to account for a variation due to differences in participants’ WM capacities, we included a WM measure in a follow-up experiment (Experiment 1b) which differed slightly from Experiment 1a. Measuring WM also provides a general assessment of cognitive ability.

Another possibility is that our shortest duration manipulation was not brief enough to have an impact on the recognition sensitivity. Considering that the duration thresholds reported in the literature as the lowest threshold for sound recognition (e.g., [16]) and pitch perception (e.g., [51]) could be around 30 milliseconds, in Experiment 1b, we replaced the 150 milliseconds condition with 30 milliseconds.

## Materials and methods

### Experiment 1b

#### Participants

26 new volunteers (mean age: 26.88 ± 3.71, 20 Females) were recruited for Experiment 1b. The data from two participants were not included in the final analyses (as one of them already participated in Exp1a, and the other had overall *d*’ scores more than 2*SD* below the group mean). All participants reported normal hearing. The selection of participants in this experiment was less strict in terms of musical training, resulting in a sample that is more representative of the general population. On average, the general musical sophistication score of the participants was 71.42 ± 23.32, which ranked at the 31st percentile according to the data norms [15]. The participants provided written informed consent and received a gift card worth 100 NOK for their participation. The experiments conformed to the Helsinki Declaration and to the national ethical guidelines for experiments with human subjects.

#### Stimuli

Stimuli were the same as in Experiment 1a, the only difference was that this time the stimuli were truncated to even shorter segments, i.e., 30 ms.

#### Procedure and design

All methodological details of the RSAP paradigm were identical to the Experiment 1a, except that the 150 ms block was replaced with the 30 ms block. As in Experiment 1a, all participants went through all four blocks and the orders of the duration blocks and targets were counterbalanced across subjects. In difference from Experiment 1a, in addition to Gold-MSI questionnaire, we also administered the Digit Span (Forward, Backward, and Sequencing subtests) test to assess WM capacity.

### Results

#### Recognition sensitivity (*d*’)

As in Experiment 1a, the overall recognition sensitivity in Experiment 1b (*d*’ = 2.14) was, too, well above the chance level.

A 4 (Duration: 30, 60, 90, 120) × 4 (Target type: voice, cello, bell, sine tone) Within-Subjects ANOVA was conducted on the *d*’ scores. The analysis showed a significant main effect of Target Type [*F*(3,69) = 11.87 *p* < 0.001, $\eta_{p}^{2}$ = 0.34]. Post-hoc contrasts indicated a better recognition for voice targets than bell targets (Mean difference = 0.345, *SE* = 0.092, *p* = 0.002), as well as better recognition for sine tones than both cello (Mean difference = 0.349, *SE* = 0.092, *p* = 0.002) and bell targets (Mean difference = 0.518, *SE*= 0.092, *p* < 0.001).

**Fig 4:**
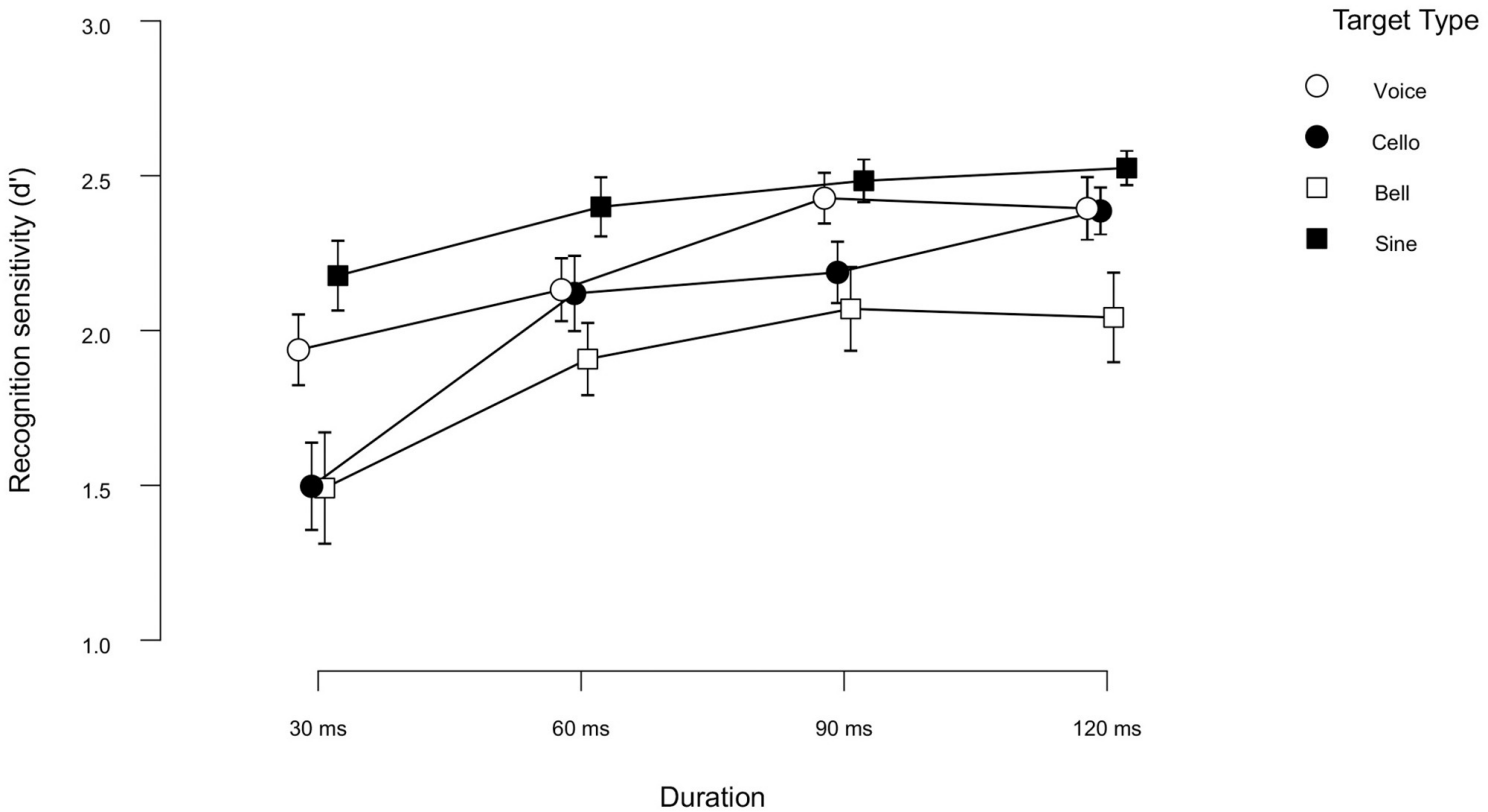
Target recognition sensitivity (*d*’) plotted as a function of stimulus duration condition (for Exp1b). Open circles, closed circles, open squares, and closed squares represent the data when the target was selected from voice, cello, bell, and sine tone categories, respectively. Error bars represent standard error of the mean.

There was also a significant main effect of Duration [*F*(3,69) = 16.34, *p* < 0.001, $\eta_{p}^{2}$ = 0.41]. Post-hoc tests revealed that the duration effect was due to lower recognition in 30 ms than in all other duration conditions (Mean differences = -0.364, -0.516, and -0.561, for 30 vs 60 ms, 30 vs 90 ms and 30 vs 120 ms respectively, all *SE*s = 0.089 and *p* < 0.001). As depicted in Fig 4, recognition performances were the lowest in the 30 ms for all targets. The recognition was the lowest was for bell (*d*’ = 1.49) and cello targets (*d*’ = 1.5) in the 30 ms condition. The interaction of Duration × Target Type, however, did not reach significance at a 5% significance level [*F*(9,207) = 1.19, *p* = 0.305, $\eta_{p}^{2}$ = 0.05].

Further Bayesian ANOVA suggested extreme evidence for the models with Target type (BF*Inc* = 8.605e+6), as well as the model with Duration (BF*Inc* = 3.119e+9). Thus, the model with Duration was around 349 times more probable than the model with Target type in explaining the data. The interaction model received strong evidence supporting the null model (BF*Inc* = 0.036), suggesting that the results were more probable to happen under the null hypothesis than the combined effect of Duration and Target Type.

**Fig 5:**
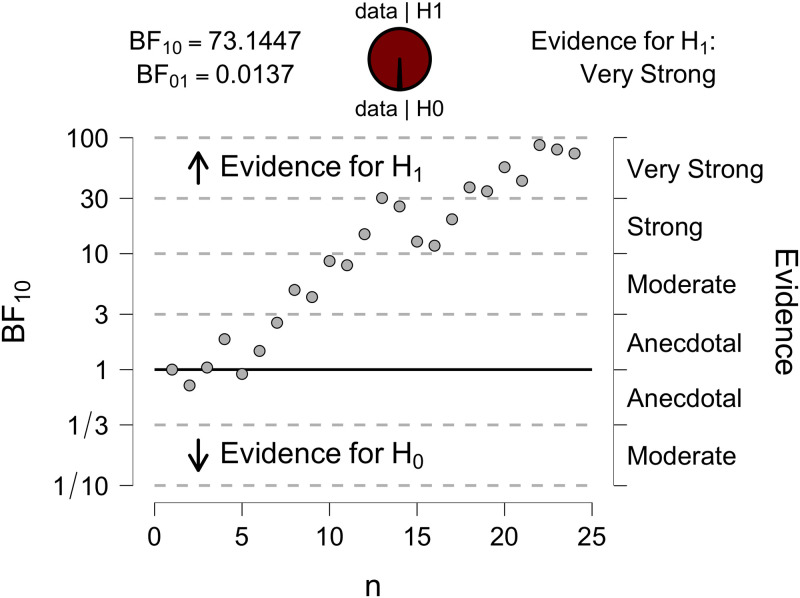
Sequential analysis with Bayesian *t*-test for the recognition of cello targets under 30 versus 60 ms conditions illustrating the development of evidence as the data accumulates. The data points for each participant are shown on the X-axis, the associated Bayes Factors are shown on the Y-axis.

Additionally, we conducted sequential analyses with Bayesian *t*-test for each target type where we tested the alternative hypothesis (*H*1) that recognition at 30 ms was not equal to recognition at 60 ms. The sequential analyses illustrate the sequential development of evidence across the study sample. Fig 5 shows that as the data accumulates, we observe a very strong evidence for the difference between recognition for cello targets under 30 versus 60 ms conditions (BF*10* = 73.14). Thus, we can conclude that recognition of cello targets suffered substantially from being truncated to 30 ms. For the rest of the target types, the evidence (supporting *H*0 in the case of voice and sine tone, and supporting *H*1 in the case of bell) was only anecdotal.

#### Reaction times

For the pre-processing of the RT data, we followed the same procedure as in Experiment 1a. Anticipation errors in Experiment 1b corresponded to 1.5% of the total trials. Overall, the participants in Experiment 1b (mean RT = 800 ms) responded faster than in those in Experiment 1a (mean RT = 908 ms) during the recognition task.

**Fig 6:**
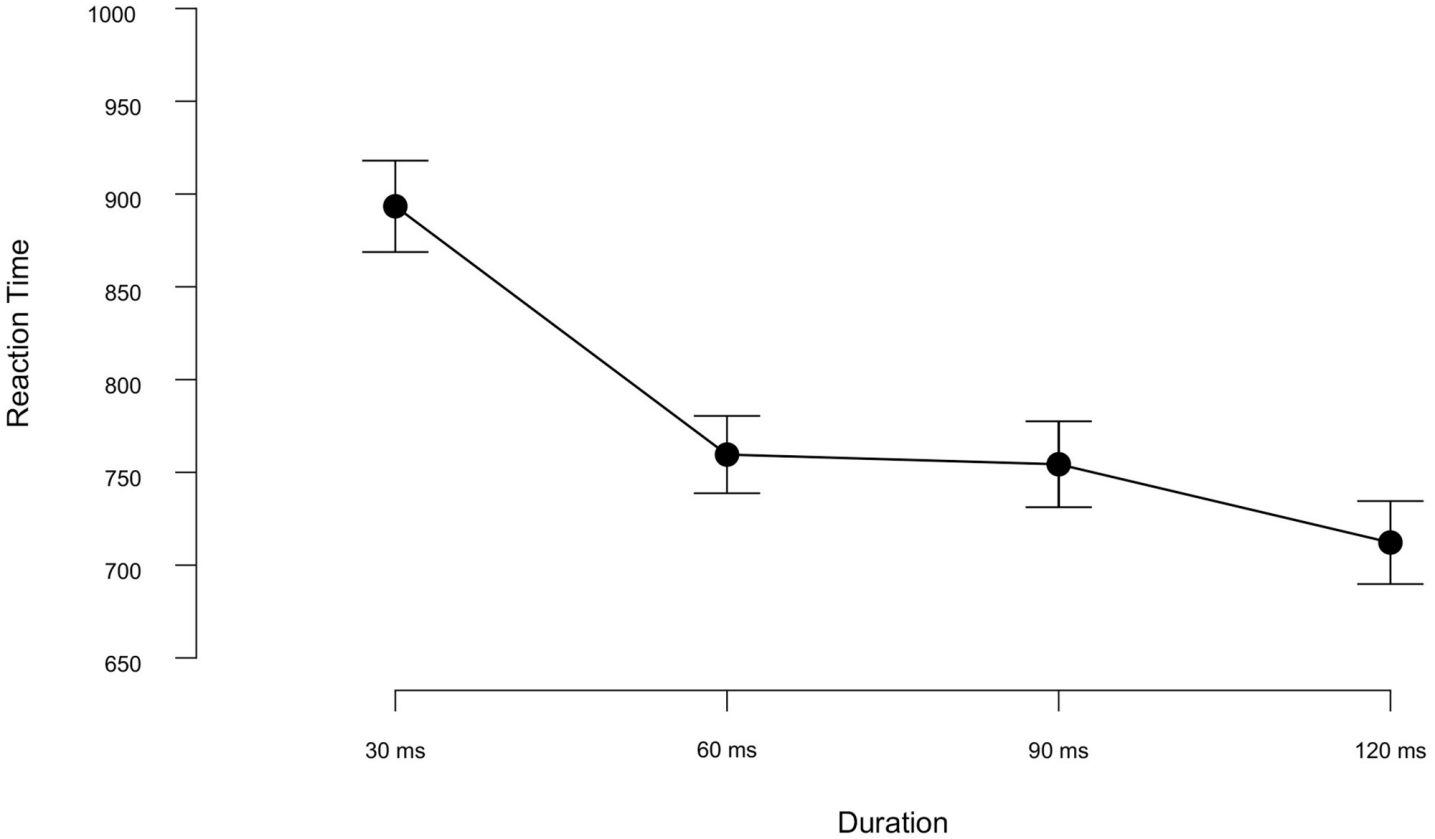
Reaction times (in ms) for target recognition as a function of stimulus duration for each item within each RSAP stream in Exp1b. Slower reaction times in the 30 ms stimulus duration condition. Error bars represent the standard error of the mean.

A 4 × 4 Repeated Measures ANOVA on the RTs indicated a significant main effect of Target Type [*F*(3,69) = 4.00, *p* = 0.01, $\eta_{p}^{2}$ = 0.148]. Post-hoc tests revealed that the participants’ responses were significantly slower for the recognition of the bell targets than of the cello targets (Mean difference = -59.96, *SE*= -110.74, *p* = 0.012). The main effect of Duration was also significant [*F*(3,69) = 4.53, *p* = 0.006, $\eta_{p}^{2}$ = 0.16]. As shown in Fig 6, the participants’ responses were the slowest in the 30 ms stimulus duration condition. Post-hoc comparisons showed that the duration contrasts with 30 ms were either statistically significant (for 30 vs 120 ms: Mean difference = 181.19, *p* = 0.005) or at the borderline of the arbitrary threshold for significance testing (for 30 vs 90 ms: Mean difference = 139.02, *p* = 0.048, for 30 vs 60 ms: Mean difference = 133.80, *p* = 0.05, all *SE*s = 52.18). Finally, the Duration × Target Type interaction effect was also statistically significant [*F*(5.375, 123.609) = 2.74, *p* = 0.02, $\eta_{p}^{2}$ = 0.107, Greenhouse-Geisser corrected], reflecting that RTs for voice targets were similar in the 30 and 60 ms conditions, while for all other targets there was a sharp increase in RTs from 60 to 30 ms duration. Thus, the speed of the recognition of voices did not suffer as much in the briefest stimulus duration as the other targets.

Further Bayesian ANOVA revealed moderate evidence in support of the null model over the model with Target Type (BF*Inc* = 0.102), extreme evidence favoring the main effect of Duration (BF*Inc* = 2.902e+6) over the null model and strong evidence against the Target Type × Duration interaction (BF*Inc* = 0.056). This means that the main effect of Duration has received the most support in explaining the RTs for recognition, which was 2.9 × 10^6^ times more probable under Duration model than the null model.

#### Level of subjective certainty

A Repeated Measures ANOVA on the participants’ subjective certainty levels indicated a significant main effect of Target Type [*F*(2.198, 50.560) = 21.59, *p* < 0.001, $\eta_{p}^{2}$ = 0.48, Greenhouse-Geisser corrected], Duration [*F*(1.690, 38.860) = 18.30, *p* < 0.001], $\eta_{p}^{2}$ = 0.44, Greenhouse-Geisser corrected] and the Duration × Target Type interaction effect [*F*(4.832, 111.144) = 5.58, *p* < 0.001, $\eta_{p}^{2}$ = 0.19, Greenhouse-Geisser corrected]. Post-hoc comparisons indicated that the participants’ subjective certainty ratings were significantly higher when the target was a sine tone than a bell (Mean difference = 0.428, *SE*= 0.057, *p* <0.001) or cello tone (Mean difference = 0.282, *SE*= 0.057, *p* <0.001). Certainty ratings were also significantly higher for the voice targets than bell targets (Mean difference = 0.301, *SE* = 0.057, *p* <0.001). The post-hoc tests also indicated that the main effect of duration was due to the contrasts with the 30 ms condition (for 30 vs 120 ms: Mean difference = -0.702; for 30 vs 90 ms: Mean difference = -0.574; for 30 vs 60 ms: Mean difference = -0.491, with *SE*= 0.102; *p* <0.001 for all contrasts).

Moreover, the comparisons for the interaction effect indicated that there was a significant drop in participants’ certainty levels in the 30 ms condition for cello (all *p* < 0.001) and for bell targets (all *p* < 0.001), while for sine tones there was not (*p* > 0.05). Finally, for the voice targets, the certainty levels in 30 ms condition did not differ significantly from that of in the 60 ms condition (Mean difference = -0.361, SE = 0.125, *p* = 0.53), but differed when contrasted with the longer durations (30 ms vs 90 ms: Mean difference = -0486, *SE*= 0.125, *p* = 0.02 30 ms vs 120 ms: -0.658, *SE*= 0.125, *p*< 0.001).

Further analyses using the Bayes Factor indicated extreme evidence in favor of the models with Target Type (BF*Inc*= 8.684e+7), Duration (BF*Inc* = 1.018e+23) and moderate evidence for the interaction of Target Type and Duration (BF*Inc* = 3.581). The model with Duration has received the most support in explaining the data, which was around 10^23^ times more probable than the null model. Thus, the subjective certainty levels were best explained by the duration manipulation and the effect appeared to be driven by the 30 ms duration condition.

#### Target position

No statistically significant difference was found between the target position 5 and 15 (*t* = 0.272, *p* = 0.79). A further Bayesian paired-samples *t*-test indicated moderate evidence (BF*01* = 4.50) in favor of the null hypothesis (i.e., it was 4.5 times more probable that recognition under the two target positions did not differ from one other).

#### Correlations

The musical sophistication scores were moderately and positively correlated with the overall Hit rates (Kendall’s tau = 0.40, *p* < 0.001) and with *d*’ scores (*r* = 0.53, *p* < 0.001).

Digit span working memory scores, however, did not correlate with the Hit rates, False alarm rates, *d*’ scores, or musical sophistication scores (all *p* >0.05). Since there was no linear correlation with the dependent variables, we did not include digit span scores as a co-variate.

**Fig 7:**
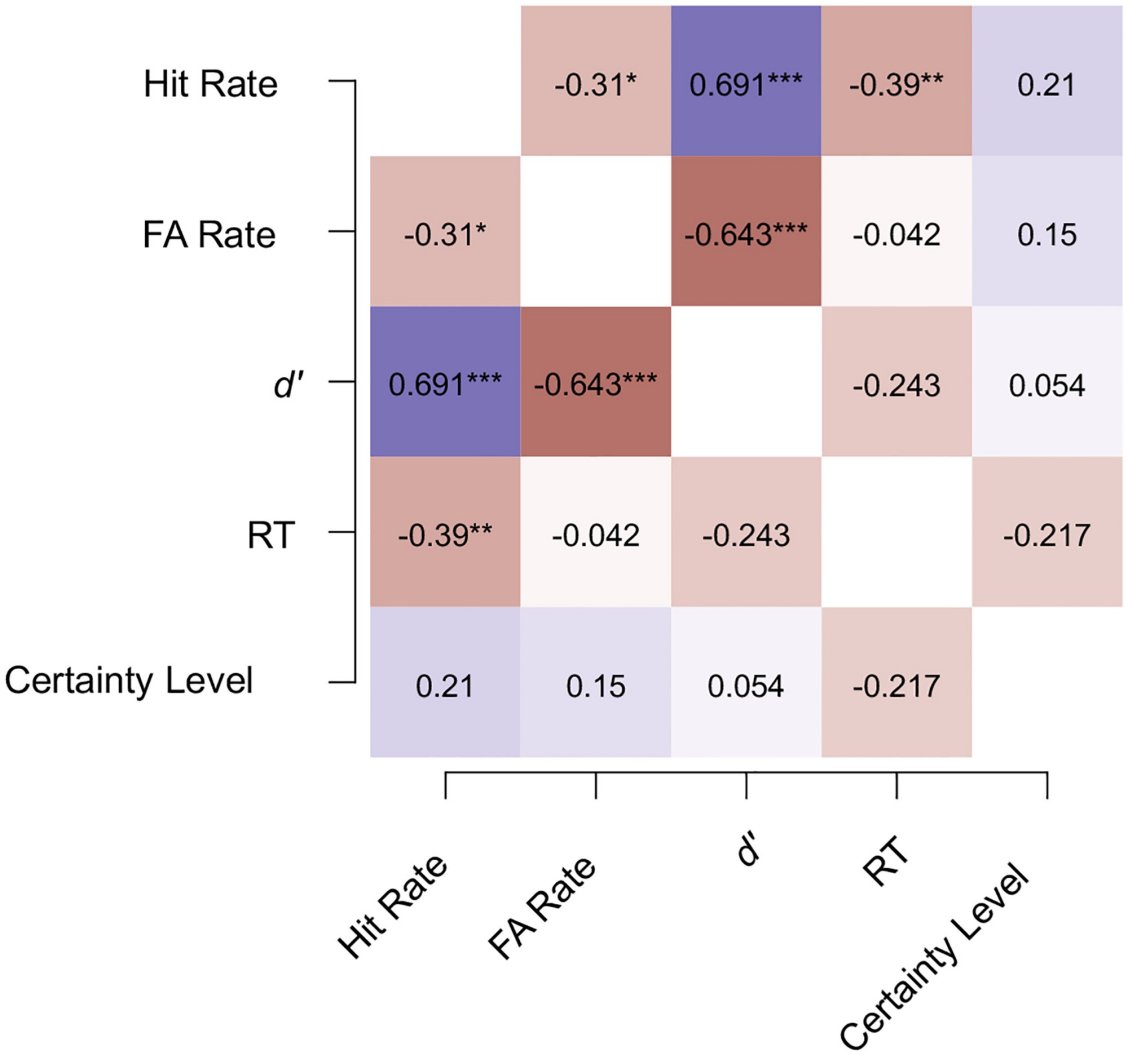
Heatmap of the correlations between the measures in Experiment 1b. The heatmap depicts Kendall’s tau correlations between the measures of Hit rate, False alarm (FA) rate, the sensitivity index (*d*’), reaction time (RT), and the subjective certainty level. Blue and red colors correspond to positive and negative correlation coefficients, respectively. The saturation of colors illustrate the size of the correlation coefficient. * *p* < 0.05, ** *p* < 0.01, ***, *p* < 0.001.

As shown in the heatmap (Fig 7), there was a moderate negative association between the Hit rates and the RTs (Kendall’s tau = -0.39, *p* = 0.009). However, the RTs did not correlate with the *d*’ scores (Kendall’s tau = -0.24, *p* = 0.10). Participants’ subjective certainty levels did not correlate with *d*’ scores (Kendall’s tau = 0.05, *p* = 0.71), Hit rates (Kendall’s tau = 0.21, *p* = 0.16), False alarm rates (Kendall’s tau = 0.15, *p* = 0.33) or with the RTs (Kendall’s tau = -0.22, *p* = 0.14).

#### Musical sophistication as a co-variate

Musical sophistication significantly predicted recognition sensitivity, *F*(1,22) = 8.65, *p* < 0.01. After controlling for the effects of musical sophistication, the effect of Target Type on recognition sensitivity remained significant [*F*(3,66) = 4.93, *p* < 0.01, $\eta_{p}^{2}$ = 0.18], indicating that sine tone targets were recognized significantly better than both cello (*p* = 0.001) and bell targets (*p* < 0.001) and that the voice targets resulted in significantly higher recognition than the bell targets (*p* = 0.001). When adjusted for the effects of musical sophistication, the main effect of duration, on the other hand, was no longer statistically significant, *F*(3,66) = 0.42, *p* = 0.74, $\eta_{p}^{2}$ = 0.02.

## Discussion

The present work consisted of two experiments examining how brief duration and sound source impact auditory recognition for target tones embedded in a rapid presentation stream. A novel aspect of these experiments was that the exploration of the association of musical sophistication and WM capacity with performance in the auditory recognition task.

The results of Experiment 1a and 1b show that people can recognize a sound source among rapidly presented environmental sounds with well above chance level sensitivity, even if they are exposed for it only a few tens of milliseconds. This suggests that just as in categorization or recognition of isolated sounds, auditory recognition within a rapid stream, too, requires very little information. Importantly, this recognition ability was observed in participants whose musical sophistication scores were lower than that of the general population.

Regarding the effects of sound duration on recognition, in Experiment 1a, we found very strong evidence suggesting that the recognition of brief sounds presented in RSAP is unlikely to be affected by the stimulus duration between 60 to 150 ms. One possible explanation of the current finding is that the sound features in the shortest stimulus duration (i.e., 60 ms) were as informative as in the longer durations and afforded more or less equally successful recognition. Notably, in Experiment 1b, a different picture emerged when we tested recognition performances under even shorter stimulus duration (i.e., 30 ms). In particular, Experiment 1b pinpointed an extreme evidence for the effect of sound duration on recognition sensitivity, with a clear decline in recognition for sounds presented for 30 ms.

This result is consistent with prior behavioral evidence using similar [16] or different paradigms [52], suggesting that the minimum duration threshold required for recognition above chance level could be around 30 ms. This is presumably because the cues for timbre were significantly more limited in the 30 ms condition than in the longer duration conditions. In other words, if it simply takes longer than 30 ms for timbre features to unfold, having less informative cues could lead to impaired recognition. In line with this interpretation, Godøy [53] describes a threshold between sub-sonic and sonic auditory domains that occurs around 20 Hz (i.e., separating features that require more or less than 50 ms to unfold). Sound features that unfold at a sub-sonic timescale (below 20 Hz) include dynamic features of timbre, pitch, and loudness, while sound features that unfold at a sonic timescale (above 20 Hz) are largely stable or very rapidly fluctuating. Alternatively, having a very limited time to encode a target sound in a rapid stream may also have resulted in impaired recognition performance, as the time required for forming a memory trace of a particular sound can be longer than the duration of that sound [54, 55]. This would reflect that the amount of time needed for encoding may not always be sufficiently long in the 30 ms condition.

One of the main findings from our study is the critical role of musical sophistication on auditory recognition. The current literature only tangentially touches upon the relationship between musicality and brief sound recognition. Often, the aspects relating to the participants’ musical sophistication are not quantified (but rather categorized) and not considered as broadly as in the present study. To the best of our knowledge, this is the first study to explore this link, using a measure that allows for capturing the differences in musical sophistication among the general population, including so-called ‘non-musicians’. In Experiment 1b, we demonstrated that variability in participants’ musical sophistication significantly predicts the recognition of brief sounds, but their WM capacity, as measured by digit span, does not. Crucially, when the differences in musical sophistication were taken into account, the effect of sound duration on auditory recognition that we discussed earlier completely disappeared. Thus, the relationship between the sound duration and recognition sensitivity appears to be spurious and attributable to differences in musical sophistication. This is in line with the findings of Experiment 1a, where we found that the duration manipulation was unlikely to affect recognition. This result is striking, given that the sample in the present study was slightly less musically sophisticated than the general population according to the norms. Our results thus support the conclusion that variability in the participants’ musical sophistication could account for the differences in minimum duration required for timbre recognition. The differences in participants’ musical sophistication, therefore, may have confounded some of the previous investigations on the brief sound recognition.

The results of the current study suggest that WM capacity may not play a substantial role in performance on the auditory recognition task. Since WM capacity was tested with a digit span task, we cannot rule out the potential role of other nonverbal components of WM (e.g., kinesthetic), which have been implicated in the processing of nonverbal sound stimuli [56]. Previous research suggests that WM for timbre relies more on the sensory trace, while WM for verbal information depends on categorical information, as the latter can be rehearsed [57]. Thus, participants might also vary in their ability to retain timbre features in sensory memory [58]. The potential contribution of these aspects of memory to auditory recognition should be explored in future research. When considering WM measure as a general assessment of cognitive ability, our results show that this does not predict individual differences in the RSAP task.

When scrutinizing the effects of sound source under the 30 ms condition, we observed that the slope of the decline for the recognition of bell and cello tones were markedly sharper than that of voices and sine tones. In other words, it seems that the recognition of the voice and sine tone targets did not suffer as much from having a very limited exposure time to the stimuli. Particularly for voices, this pattern of results lends further support to the idea of a processing advantage for voices. An advantage for voices over the instrumental and/or environmental sounds has been reported in numerous behavioral studies using brief sound stimuli [6, 13, 16, 59]. Furthermore, neuroscientific evidence also suggests greater neural selectivity for voices in human auditory cortex [60–65].

Under the assumption that stimuli that conveys more information could be more efficiently encoded and maintained in memory [66, 67], these results could also indicate that the voice and sine tone targets were richer in information even under the brief duration constraints than the other targets. This explanation may be reasonable for the recognition advantage for very brief voice targets (as voices can convey a wealth of information, such as gender, emotional state, and age [68]), but is at odds with explaining our findings for the sine tones (as it is unclear to us in what ways sine tones could be loaded as such). Indeed, sine tones are argued as neither rich in information nor perceptually sophisticated [69]. Better recognition for the sine tones could therefore simply be as a result of the simplicity of the sine tones. To put in other words, a more complex stimuli would have more to lose from the truncation of the auditory signal. It is also possible that their simplicity within the stimulus set made them more distinct. Given the target sounds were presented amongst the distracters, the task places a high demand on the discriminability against the distracters. Hence, the degree of similarity in the acoustic features of the target and the environmental sounds could make it more or less difficult to segregate a certain target stimulus. Indeed, our descriptive analysis (S1 Appendix in S1 File) showed that the acoustic features (particularly, entropy, spectral spread and brightness) of sine tone targets, but not the voices, differed substantially from the distracters. Thus, the difference in these low-level features between distracters and sine tones provides an explanation for better recognition for sine tones in the present experiments.

With regard to the impact of sound source on auditory recognition, in both experiments, sine tones were better recognized than bell tones. In difference from Experiment 1a, the influence of sound source on recognition in Experiment 1b was also partly due to the better recognition for sine tone targets than cello targets and for voice targets than bell targets. Importantly, the effect of sound source on recognition remained significant even after controlling for the differences in musical sophistication (Experiment 1b). In both experiments, the largest difference was observed between the recognition of the sine tone and the bell targets, with better recognition for the sine tones. This is likely, at least partly, due to the perceptual representations of timbre, encompassing a variety of acoustic properties differing between a sine tone and a bell sound. For example, impaired recognition for the bell sounds can be attributable to the perceived pitch fluctuations, or inharmonicity, of the bell sounds. Hence, making the bell sounds more ‘noisy’ than the other sound sources, and this is said to be “nearly always very important for the perception of timbre” [70].

Other than the differences in simple acoustics, cognitive and semantic differences can also account for the observed difference in the recognition performance for sine tone and bell targets. For example, the distracters could have interfered with the auditory trace of the bell target more than they do with the other target categories, because of the bell sounds being semantically similar to the environmental sounds (which served as distracters in the present study). This interference could be either as a result of the content (i.e., interference-by-content, that is, memory distruption induced by the presence of distracters in memory similar in identity to the target item) or the process (i.e., interference-by-process, by a conflict a processes of seriation during the rehearsal of target items and the pre-attentive encoding of the order of the distracter stream, [71]). In our case, the former is a more likely explanation, given that despite the serial presentation of the sounds, there was only one target to report, hence, no temporal ordering of the targets was required.

Though we do not know precisely which information our participants may have relied more or less on during the recognition task, the experiments by Gregg and Samuel [72] provide evidence that although auditory representations are likely to include both semantic and acoustic information, listeners rely more on the semantics and that the semantic identity information is encoded in rich detail as compared to the acoustic information. It is also worth mentioning that the recognition in the context of our experimental paradigm is likely to encompass several meanings as described by McAdams [2]: corresponding to something that has been heard earlier (i.e., during the presentation phase), something that is accompanied by a sense of familiarity, making sense of the source identity, an understanding of what the source signifies for the listener. In order to determine the separate effects for each aspect, further research on timbre recognition is needed.

Recent evidence by Siedenburg and McAdams [39] suggests a salient role of long-term familiarity with sound sources in timbre recognition. The authors explain that familiar sounds offer more affordances for ‘deep’ encoding of timbre. At the time of encoding, deep processing occurs when novel information is integrated with pre-existing schematic knowledge [73]. Deep encoding for familiar timbres involves, in addition to auditory representation, some level of activation in semantic, visual and sensorimotor networks, which can bind with the associated auditory traces and lead to better recognition [39]. Simply put, according to this view, the more a timbre can afford this kind of multilayered and deep encoding, the more robustly it will be recognized. With this in mind, participants’ long-term familiarity with the timbres in the present study could have enhanced their encoding, and hence their recognition. Despite the fast presentation rates and the brief sound duration manipulation that put significant demands on the attentional resources required at the time of encoding, timbre recognition for brief sounds in the present study was well above chance. It would be very interesting to explore if this would be the case for the non-familiar timbres, too. Future studies could explore the relationship between familiarity in the context of timbre recognition for brief sounds further.

It is conceivable that with testing even briefer sound durations, we would have observed a significant effect of duration on timbre recognition, even after controlling for musical sophistication. As mentioned earlier, due to the different nature of the paradigms and study designs, the task in the current experiments was expected to be somewhat more difficult than in the previous investigations. In retrospect, testing even briefer durations would help determine the minimum duration that allows for successful timbre recognition in the context of our study. While it is not possible at this time to be precise about the threshold, the durations tested here are still well within the limits used in auditory variant of the attentional blink [19] studies, which also typically employ the rapid presentation stream paradigm.

Finally, the RT findings were somewhat inconsistent compared to the findings obtained from the recognition sensitivity measure. In Experiment 1a, we observed that the results conclusively supported the null-effect over the effects of duration and target type on the RTs. This means that none of the manipulations in Experiment 1a seem to influence the participants’ response times, which is in contrast to the results obtained with the *d*’ parameter. In Experiment 1b, on the other hand, while both the duration and the target type models received support over the null hypothesis, the model with the duration explained our RT data the best. The RT results in this experiment were more consistent with the recognition sensitivity findings.

Regarding the RT results, we would like to point out that due to the device we used (i.e., keyboard) some delay in the reported RTs is expected. However this delay was very small (i.e., an average of 14.82 ms and standard deviation of 3 ms) and the RT differences we reported (around 200 ms) well exceeds the delay of the device. Second, although it may seem straightforward to relate recognition sensitivity and RTs to a common underlying process, given that the task in the present study was data-limited in nature (i.e., as a consequence of the brief duration manipulation, limiting both the time that the stimulus is available for processing and the quality of the information within each stimulus), the two measures may not necessarily reflect the same underlying processes (see [74]). The finding that either no correlation (Experiment 1a) or weak negative correlation (Experiment 1b) was observed between the RTs and the *d*’ parameters, could support the argument that they could tap into different processes, as the results do not converge. Previously it is argued that under the data-limited conditions, the findings from the recognition accuracy might be more sensitive to capturing the early perceptual processes while the RT results might reflect more the later response interference effects [74]. However, in the context of our study, it is difficult to make a clear cut separation of these two processes. The results obtained from both measures could still be partially attributable to both processes, rather than reflecting the separate effects of each. This is because the study instructions included requirements for both the accuracy and the speed of the response. Note, however, as an attempt to increase the validity of the recognition sensitivity measure, our instructions included a slight emphasis on the accuracy over the speed (i.e., requiring participants to respond as fast as possible, but without compromising accuracy). Future studies could benefit from having a better control for these factors.

## Conclusions

Taken together, the present findings extend the previous conclusions by suggesting that while the successful recognition of a single sound presented within a rapid auditory stream requires very little information, the minimum duration required for recognition of different sound sources could be different. Critically, though it seems that auditory recognition is significantly impaired at the 30 ms stimulus duration, when controlling for the variability in the participants’ musical sophistication, the manipulations of brief sound duration ceases to affect recognition.

## Supporting information

S1 Dataset Data from experiment 1a. (TXT)

S2 Dataset Data from experiment 1b. (TXT)

S1 File (PDF)
