# Supplementary material for: Recognition of brief sounds in rapid serial auditory presentation
Source: PLoS One. 2023 Apr 13;18(4):e0284396. doi: 10.1371/journal.pone.0284396 (PMC10101377; doi:10.1371/journal.pone.0284396)
Supplement: S1 File — (PDF) [file pone.0284396.s003.pdf]

Supplementary Material

M. Akça, J. K. Vuoskoski, B. Laeng, and L. Bishop (2022)

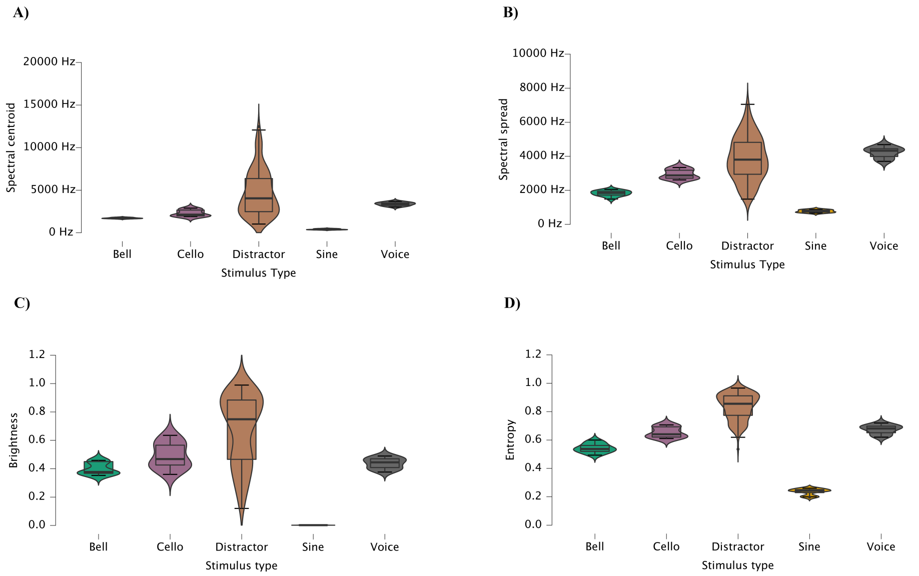

S1 Appendix Distributions of timbre-related spectral features within the different stimulus categories. Spectral features of the stimuli were extracted using the MIRtoolbox for Matlab (Lartillot, Toiviainen, Eerola, 2008; Lartillot, 2019). Based on the MIRtoolbox manual and Alluri et al. (2012), we provide brief explanations of each feature: Spectral centroid refers to the geometric centre on the frequency scale of the amplitude spectrum. Spectral spread (range: 0 - 1) is the standard deviation of the spectrum. Brightness (i.e., high energy/low energy ratio; refers to the ratio of energy content below and above 1500 Hz. Spectral entropy (range: 0 - 1) is the relative Shannon entropy (1948) of the spectrum and indicates whether the spectrum contains predominant peaks (e.g., a single sine tone has minimal entropy and white noise maximal).

Bayes factor	Evidence category
> 100	Extreme evidence for H1
$30 - 100$	Very strong evidence for H1
$10 - 30$	Strong evidence for H1
$3 - 10$	Moderate evidence for H1
$1 - 3$	Anecdotal evidence for H1
1	No evidence
$1/3 - 1$	Anecdotal evidence for H0
$1/10 - 1/3$	Moderate evidence for H0
$1/30 - 1/10$	Strong evidence for H0
$1/100 - 1/30$	Very strong evidence for H0
$< 1/100$	Extreme evidence for H0

S2 Appendix Bayesian evidence ratio interpretations. Interpretations suggested by Lee and Wagenmakers (2013); adjusted from Jeffreys (1961).
